# Multidimensional integrative analysis uncovers driver candidates and biomarkers in penile carcinoma

**DOI:** 10.1038/s41598-017-06659-1

**Published:** 2017-07-27

**Authors:** Fabio Albuquerque Marchi, David Correa Martins, Mateus Camargo Barros-Filho, Hellen Kuasne, Ariane Fidelis Busso Lopes, Helena Brentani, Jose Carlos Souza Trindade Filho, Gustavo Cardoso Guimarães, Eliney F. Faria, Cristovam Scapulatempo-Neto, Ademar Lopes, Silvia Regina Rogatto

**Affiliations:** 1 A.C.Camargo Cancer Center, São Paulo, SP Brazil; 2 Center of Mathematics, Computing and Cognition, Federal University of ABC – UFABC, Santo André, SP Brazil; 3 Brazilian Biosciences National Laboratory (LNBio), Campinas, SP Brazil; 4 Department of Psychiatry, Medical School, University of Sao Paulo – USP, São Paulo, SP Brazil; 5 Department of Urology, Faculty of Medicine, Sao Paulo State University - UNESP, Botucatu, SP Brazil; 6 Department of Urology, Barretos Cancer Hospital, Barretos, São Paulo, Brazil; 7 Molecular Oncology Research Center, Barretos Cancer Hospital, Barretos, SP Brazil; 8 Department of Clinical Genetics, Vejle Hospital and Institute of Regional Health, University of Southern Denmark, Odense, Denmark

## Abstract

Molecular data generation and their combination in penile carcinomas (PeCa), a significant public health problem in poor and underdeveloped countries, remain virtually unexplored. An integrativemethodology combin ing genome-wide copy number alteration, DNA methylation, miRNA and mRNA expression analysis was performed in a set of 20 usual PeCa. The well-ranked 16 driver candidates harboring genomic alterations and regulated by a set of miRNAs, including hsa-miR-31, hsa-miR-34a and hsa-miR-130b, were significantly associated with over-represented pathways in cancer, such as immune-inflammatory system, apoptosis and cell cycle. Modules of co-expressed genes generated from expression matrix were associated with driver candidates and classified according to the over-representation of passengers, thus suggesting an alteration of the pathway dynamics during the carcinogenesis. This association resulted in 10 top driver candidates (*AR, BIRC5, DNMT3B, ERBB4, FGFR1, PML, PPARG, RB1, TNFSF10* and *STAT1*) selected and confirmed as altered in an independent set of 33 PeCa samples. In addition to the potential driver genes herein described, shorter overall survival was associated with *BIRC5* and *DNMT3B* overexpression (log-rank test, P = 0.026 and P = 0.002, respectively) highlighting its potential as novel prognostic marker for penile cancer.

## Introduction

Data integration has emerged as a promising mechanism for the association of events affecting biological pathways and tumor development^1^. Due to the high mutational burden of cancer genomes, the distinction between driver and passenger genes is a challenge^2^. Passenger mutations were believed to not affect cell growth and to be accumulated during tumor progression. However, more recently, the accumulation of deleterious passengers has been suggested as being associated with carcinogenesis, leading to an immune response and cellular stress, as well as contributing to therapy-resistance^3, 4^.

The identification of these biomarkers is hampered by genome complexity and limited investigation at a molecular level, which does not allow a broad overview of the different mechanisms involved in gene activity^5^. In order to overcome this issue, the combination of different molecular alterations in a comprehensive manner has been explored as a mechanism to reveal potential gene candidates associated with targeted pathways by therapeutic agents^6^.

Recent initiatives, such as TCGA (The Cancer Genome Atlas) and ICGC (International Cancer Genome Consortium), rendered novel insights on cancer system biology compared with isolated events^7^. At the same time, the combination of heterogeneous datasets is particularly difficult to analyze. This encouraged initiatives to design a broad-spectrum of integrative analysis^6^. Module-based approaches have emerged as an efficient mechanism to reconstruct modules of co-regulated genes and their regulatory programs^8^. This methodology has been widely used to explore various biological contexts in cancer studies^9, 10^. Although novel targeted-genes for cancer therapy have been described, there is a lack of studies generating and combining molecular data of penile carcinomas.

Penile carcinoma (PeCa) is a rare genitourinary malignancy in developed countries, with an incidence of 0.2 per 100,000 men in the United States and Europe^11, 12^ and 2.9 to 6.8 cases per 100,000 in the Brazilian population^13^. The risk factors described in PeCa include phimosis with chronic inflammation, poor hygiene, smoking, low socioeconomic status, number of sexual partners, a history of genital warts and/or other sexually transmitted diseases^14^. Approximately 40% of PeCa are HPV positive, however, the impact of high-risk HPV in the prognosis has not been clarified^15^. Recently, in a large international study applied in 25 countries, HPV positivity was described in 33% of PeCa (N = 1010) and 87% of precancerous lesions (N = 85)^16^.

Several prognostic factors have been established for PeCa patients, while regional inguinal lymph node involvement remains the most important predictor of an unfavorable prognosis^17^. Patients with locally advanced penile squamous cell carcinoma and lymph node metastasis are submitted to total or partial penile amputation, followed by primary chemotherapy or radiotherapy^18^. In a recent review, Burnett *et al*.^19^ presented different surgical options available for penile-preservation at early stages and the need for patient monitoring. Besides having a curative effect even in the most advanced diseases, these surgical procedures results in a significant burden of social and psychological impact for the patient, highlighting the importance of identifying molecular markers for penile cancer therapy^20^.

Previously, we reported an association between genomic alterations involving losses of 3p21.1-p14.3 and gains of 3q25.31-q29 with reduced cancer-specific and disease-free survival^16^. *DLC1* and *PPARG* losses were also associated with worse prognosis. By integrating methylome and gene expression data, we described a panel of 54 genes with inverse correlation (including *TWIST1*, *RSOP2*, *SOX3*, *SOX17*, *PROM1*, *OTX2*, *HOXA3* and *MEIS1*), pointing out driver epigenetic events associated with dysregulated pathways in PeCa, such as stem cells, Wnt/β-catenin signaling and cell cycle^21^. More recently, by assessing 23 PeCa patients we identified a high sensitivity and specificity of *PPARG*, *MMP1* and *MMP12* and hsa-miR-31-5p, hsa-miR-223-3p and hsa-miR-224-5p to distinguish penile tumors from normal tissue^22^. Next generation sequencing studies in penile carcinomas revealed the involvement of well-described genes, such as *EGFR*, *PIK3CA*, *TP53* and *CCND1* ^23–25^ and dysregulated miRNAs^26^, all associated with cancer signaling pathways.

In this study, we used a module-based integrative methodology to identify and contextualize driver genes in pathways involved in penile carcinogenesis aiming to explore genome-wide copy number alteration (CNA), DNA methylation, miRNA and gene expression (GE) data. To our knowledge, this is the first study with a multidimensional integrative approach using four molecular levels to identify novel driver candidates with potential therapeutic application.

## Results

### Integrative analysis to uncover candidate genes involved in PeCa development and progression

The first step of the integrative analysis resulted in 389 genes with varying score between 4.11 and 101.56, with expression levels regulated by at least two other molecular mechanisms. A cutoff of 48.72 was considered to separate 47 potential driver candidate genes used in the module-based analysis (Table 1) and 342 passenger candidates (Supplementary Table S1). Seventeen of 47 (36%) genes were mapped in chromosome 3, followed by chromosomes 2 (6/47) and 8 (6/47). The genomic alterations included 34 losses and 13 gains. Although the 47 driver candidates presented significant copy number alterations, with frequency varying from 35% to 90% of cases, 17 (36%) of them presented expression levels regulated by methylation, with a predominance of hypermethylation (16 of 47 genes). Fifty differentially expressed miRNAs were associated with the regulation of 47 driver candidates (Supplementary Table S2). hsa-miR-34a and hsa-miR-130b overexpression were predicted as regulators of the higher number of downexpressed driver candidates (17 and 16, respectively). Interestingly, 17 of 47 driver candidates (including 26 miRNAs) showed expression levels regulated by three molecular mechanisms investigated in this study (i.e. copy number alteration, methylation and miRNA).

**Table 1:** Forty-seven candidate genes selected in the first step of the integrative analysis.

Gene	Chromosome	LR CNA	FC Gene Expression	FC Methylation	FC miRNA	Score
*IL17RD*	3	−0.33	−2.71	1.62	8.03	101.56
*BCL2*	18	−0.42	−2.70	1.45	7.53	96.73
*IGFBP5*	2	−0.35	−3.13	1.60	6.50	92.68
*FOXP1*	3	−0.51	−1.84	1.70	6.98	88.17
*MTUS1*	8	−0.39	−1.37	1.94	6.85	84.39
*SOX7*	8	−0.57	−1.14	1.97	6.64	82.52
*AR*	X	−0.35	−4.84	−1.38	8.01	79.20
*MMP9*	20	0.31	4.25	−1.44	−3.87	79.00
*GNG7*	19	−0.27	−2.30	1.53	5.66	78.00
*SORCS3*	10	−0.54	−4.27	−1.40	7.74	75.31
*POLQ*	3	0.73	3.01	0	−8.58	73.95
*PPARG*	3	−0.36	−3.58	1.40	3.49	70.65
*ERBB4*	2	−0.50	−3.23	0	7.96	70.11
*MMP1*	11	0.57	5.44	0	−5.56	69.39
*SVIL*	10	−0.44	−2.05	1.42	4.59	68.01
*TACC1*	8	−0.43	−2.87	0	7.69	65.98
*SORBS1*	10	−0.26	−5.06	0	5.58	65.38
*OGG1*	3	−0.38	−1.29	1.21	5.03	63.29
*TFRC*	3	1.40	2.31	0	−6.80	63.07
*RFX2*	19	−0.28	−2.45	1.71	3.44	63.00
*CUL3*	2	−0.50	−2.16	0	7.75	62.49
*ZEB1*	10	−0.46	−1.81	0	8.01	61.67
*PML*	15	0.39	1.43	0	−8.31	60.78
*BIRC5*	17	0.33	3.74	0	−5.94	60.07
*ITPR1*	3	−0.49	−2.57	0	6.72	58.69
*CTDSP1*	2	−0.49	−1.41	2.00	3.34	57.94
*PAX3*	2	−0.41	−3.39	−1.52	5.68	56.84
*DNMT3B*	20	0.39	1.39	0	7.35	54.77
*GSK3B*	3	0.81	1.26	1.45	−6.99	54.38
*RB1*	13	0.97	0.92	2.12	−7.11	54.03
*ARHGEF12*	11	−0.63	−0.98	0	7.22	52.94
*CADM1*	11	−0.78	−2.27	−1.26	5.71	52.59
*PPP2CB*	8	−0.63	−0.86	1.40	3.68	52.50
*STAT1*	2	0.26	1.94	1.75	−6.53	52.39
*LRIG1*	3	−0.55	−1.92	0	6.25	52.31
*FGFR2*	10	−0.38	−1.05	1.37	3.73	52.24
*TNFSF10*	3	0.40	1.07	0	−7.23	52.23
*ADAMTS9*	3	−0.52	−1.89	−1.37	6.27	52.12
*MKRN2*	3	−0.45	−1.10	0	7.10	51.96
*TGFBR2*	3	−0.72	−1.31	0	6.62	51.88
*ITGA9*	3	−0.42	−2.52	0	5.70	51.80
*FGFR1*	8	−0.60	−3.08	0	4.91	51.54
*RANBP3*	19	−0.43	−0.92	1.43	3.63	51.34
*MYOM2*	8	−0.37	−1.82	1.48	2.74	51.26
*CADM2*	3	−0.42	−1.55	0	6.54	51.04
*SERP1*	3	0.42	1.22	1.63	−6.78	50.50
*FNDC3B*	3	0.63	1.20	1.34	−6.37	49.21

Scores higher than 48.72 (median among the lowest: 4.11 and highest: 101.56 scores) were used to select potential driver genes among the 389 candidates. Transcript levels were regulated by three molecular mechanisms in 32% (15 of 47) of the genes. Significant alterations considered in this step were identified in more than 20% of the patients. LR: Log ratio; FC: Log transformed Fold-Change; CNA: Copy Number Alteration.

### Modules identification and assignment of driver candidates

A matrix with 4,607 differentially expressed genes was submitted to clustering analysis using a Gibbs sampling algorithm^27^, which generated 418 modules composed by 3,322 genes. Modules with less than five genes were removed, resulting in 113 modules and 2,846 genes (approximately 25 genes per module). The previously identified 47 driver candidates were assigned as regulators of the 113 selected modules, resulting in 6,561 driver-module associations that were ranked by score. The top 1% high-scoring association was selected for detailed analysis. Modules with less than 10% of passenger genes were filtered out, resulting in 19 modules associated with 16 driver candidates (*STAT1*, *BIRC5*, *TNFSF10*, *PML*, *FGFR1*, *DNMT3B*, *ERBB4*, *RB1*, *AR*, *PPARG*, *SOX7*, *BCL2*, *IGFBP5*, *PAX3*, *CUL3* and *RANBP3*) (Table 2). Modules 55 (*RB1* and *IGFBP5*), 49 (*FGFR1* and *BIRC5*) and 97 (*PPARG* and *AR*) were predicted to be regulated by two driver candidates. A median of 41 genes, including 12 passengers, was detected in each module. The modules 52 (13/25), 73 (7/14), 92 (6/11), 95 (7/14) and 97 (8/16) presented more than 50% of passenger genes. The highest score was detected in module 38 (Score = 119.19), which is regulated by *STAT1* gene (Table 2).

**Table 2:** Driver candidates identified in the module-based analysis.

Regulator	Module	Assignment Score	Total number of genes/module	Passengers/module	MSigDB categories	Drug(s)
*STAT1*	38	119.19	28	9 (32%)	TF	
*BIRC5*	16	109.21	38	14 (37%)		
*TNFSF10*	48	68.60	70	17 (24%)	CDM, CGF	
*PML*	85	67.12	31	10 (32%)	O, TF, TCG	D
*FGFR1*	49	66.73	39	10 (26%)	O, CDM, PK, TCG	D
*BIRC5*	49	65.83	39	10 (26%)		
*DNMT3B*	1	65.24	71	22 (31%)		
*ERBB4*	10	64.55	33	12 (36%)	PK	D
*RB1*	55	64.22	37	11 (30%)	TS, TF	
*AR*	52	63.89	27	15 (56%)	TF	D
*PPARG*	97	63.84	17	9 (53%)	O, TF, TCG	D
*BIRC5*	102	56.81	174	20 (11%)		
*BCL2*	2	53.36	45	10 (22%)	O, TCG	D
*BCL2*	40	52.92	68	10 (15%)	O, TCG	D
*IGFBP5*	55	49.01	37	11 (30%)		
*SOX7*	6	44.23	43	18 (42%)		
*AR*	97	42.66	17	9 (53%)	TF	D
*PAX3*	73	42.20	15	8 (53%)	O, TF, HP, TCG	
*STAT1*	72	39.37	17	8 (47%)	TF	
*BIRC5*	34	37.53	29	12 (41%)		
*CUL3*	95	31.17	14	7 (50%)		
*RANBP3*	11	30.11	45	15 (33%)		

Sixteen genes were assigned as regulators of 19 modules, with scores ranging from 30.11 to 119.19. Modules with less than 10% of passenger genes were removed. Eight genes were described as drug targets and 62.5% (10/16) were annotated in at least one biological function using MSigDB categories. Modules 49, 55 and 97 were associated with two driver candidates. D: Drug target; TF: Transcription Factor; CDM: Cell Differentiation Markers; CGF: Cytokines and Growth Factor; O: Oncogenes; TCG: Translocated Cancer Genes; PK: Protein Kinase; TS: Tumor Suppressor; HP: Homeodomain Protein.

### *In silico* enrichment of biological process and pathways of the driver-module association

Nineteen modules with high scores were submitted to an enrichment analysis (GSEA, P < 0.05), revealing an association with 843 GO categories and 42 pathways (KEGG and Reactome). The majority of these modules was associated with cancer-related pathways. Biological processes associated with immune system, signal transduction, transcription factor activity, carbohydrate metabolism and cytoskeleton were the most significant categories in modules 2, 11, 48 and 102 (P-value varying from 1.14 × 10^−8^ to 3.31 × 10^−14^) (Supplementary Table S3). Pathways involved with tumor development, including homeostasis, immune system and apoptosis were predominantly enriched for modules 2 (10 pathways), 48 (5 pathways) and 55 (5 pathways) (Supplementary Table S4).

Using the Molecular Signatures Database (MSigDB), 16 driver candidates were categorized in cancer-associated groups, such as cytokines and growth factors, transcription factors, homeodomain proteins, cell differentiation markers, protein kinases, translocated cancer genes, and also oncogenes and tumor suppressors. Ten (*TNFSF10, FGFR1, PAX3, PML, PPARG, BCL2, ERBB4, AR, STAT1* and *RB1*) of 16 genes were annotated in at least one of these biological functions. Moreover, *PML, ERBB4, AR, PPARG, BCL2* and *FGFR1* were identified as drug-targets (DrugBank database) (Table 2).

**Figure 1:**
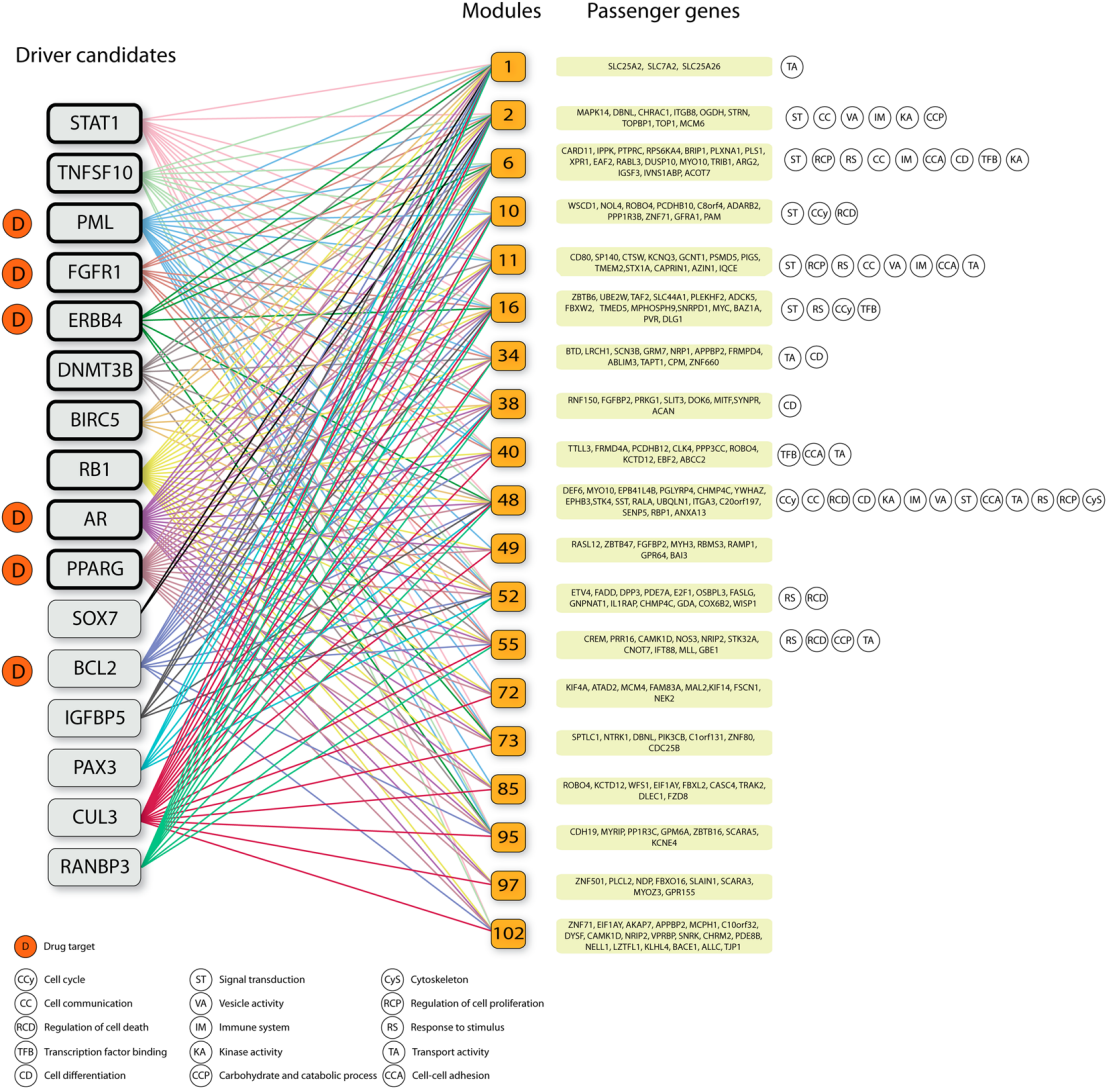
Protein-protein interaction (PPI) network illustrating the connectivity betwen 16 driver candidates and 19 modules. All driver candidates showed association with at least two modules, indicating a possible interconnection among driver genes activity in the regulation of an important biological process related with cancer development. *RB1* and *AR* genes presented the highest connectivity with modules (19 edges), followed by *CUL3* and *PPARG* (18 edges). Passenger genes and significant GO categories associated with modules were illustrated. Modules 48 and 102 presented associations with the largest number of GO categories (12 and 10, respectively). Transcription levels of the driver candidates selected and confirmed by RT-qPCR were highlighted with black outline in the rounded rectangle.

A protein-protein interaction (PPI) analysis revealed an association among the 16 driver candidates and 19 modules. *RB1* and *AR* genes presented the higher connectivity degree with modules (19 edges), followed by *CUL3* and *PPARG* (18 edges). Passenger genes were over-represented in all modules and the enrichment analysis revealed significant gene ontology categories associated with well-connected modules, such as module 6 (17 passengers and 12 GO categories), 48 (16 passengers and 9 GO categories) and 102 (19 passengers and 10 GO categories) (Fig. 1).

### Cross-study validation test to identify PeCa driver candidates in other SCC histological subtypes

Transcriptomic profile of the selected 16 driver candidates identified in the set of PeCa was compared to the expression profile of head and neck (460 T and 44 N), cervical (19 T and 3 N) and lung squamous cell carcinomas (501 T and 51 N) using data retrieved from TCGA. As shown in the online Supplementary Table S5, 15 genes displayed significant differential expression in at least one tumor type (Limma, P < 0.05). Although not significant, *RB1* overexpression was found in head and neck carcinomas.

### Gene expression pattern of driver candidates by RT-qPCR

**Figure 2:**
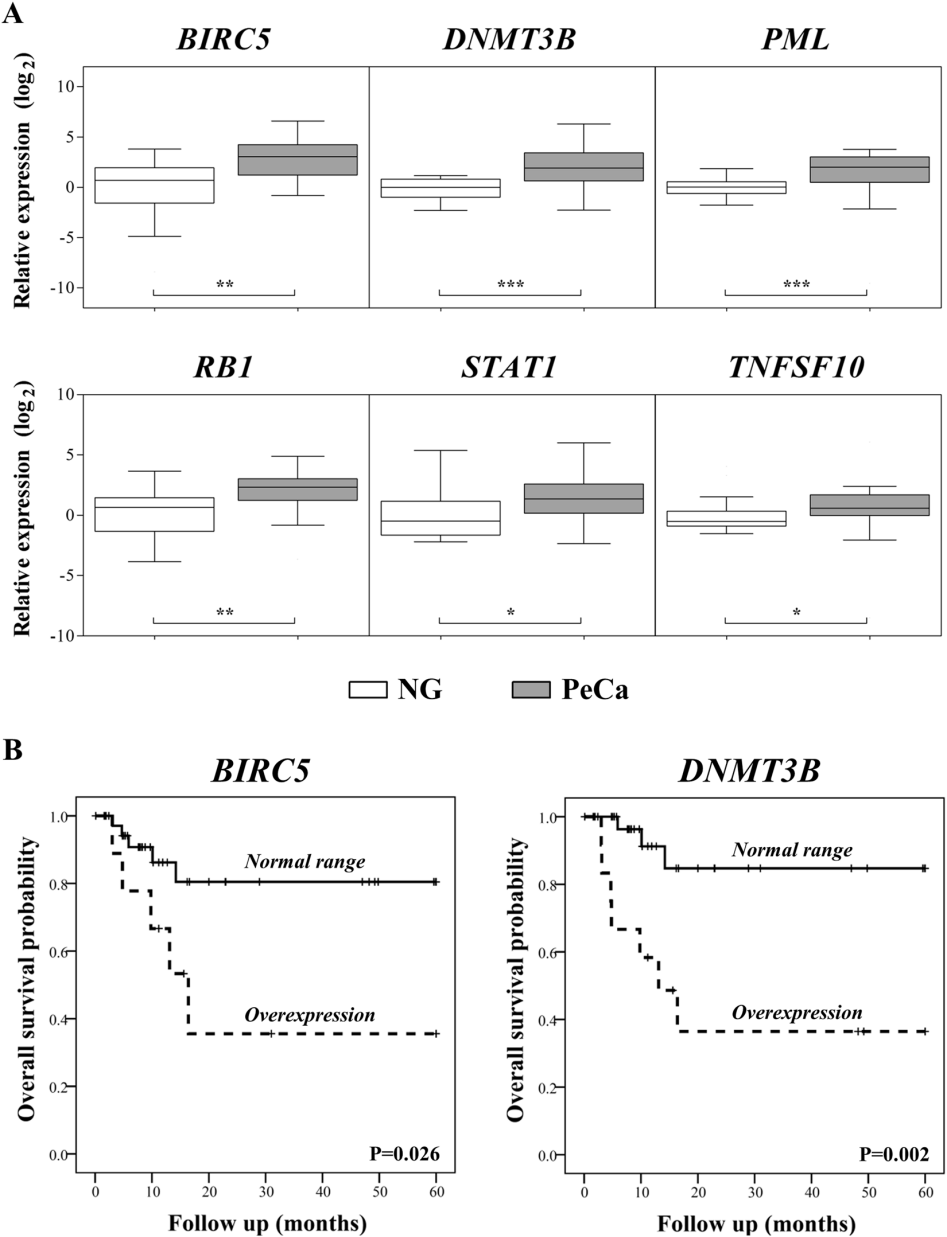
(**A**) Boxplot representation of the RT-qPCR data performed in the microarray-independent set of samples, showing expected significant results for all assessed transcripts (Mann Whitney test *P < 0.05; **P < 0.01; ***P < 0.001). (**B**) Overall survival curves of *BIRC5* and *DNMT3B*, demonstrating a significant short overall survival (log rank test P < 0.05) in patients who exhibit overexpression of these genes. **Legend:** NG: Normal glans; PeCa: Penile Carcinoma.

The cutoff of 44.54, which is the median value between the lowest (30.11) and highest (119.19) score, was used to rank the 22 associations, including 16 driver candidates and 19 modules. Ten selected transcripts were evaluated by RT-qPCR in the same set of 20 PeCa used in the arrays and in 33 cases selected for data validation. Significant overexpression of *BIRC5, DNMT3B, PML, RB1, STAT1* and *TNFSF10* genes was confirmed as altered (Fig. 2A; Supplementary Table S6). Downexpression of *AR, PPARG, ERBB4* and *FGFR1* was previously confirmed in the same set of PeCa samples used in this study^21^. Of note, *BIRC5* and *DNMT3B* overexpression were associated with shorter overall survival (log-rank test, P = 0.026 and P = 0.002, respectively) (Fig. 2B). Although our set of patients includes a limited number of death events(11), the multivariate analysis confirmed *DNMT3B* as significantly associated with shorter overall survival, revealing its potential as a prognostic marker in PeCa (Cox Regression, P = 0.015 OR = 5.4 CI 1.4-21.2) (Supplementary Table S7).

## Discussion

Studies implementing and exploring integrative approaches have unveiled therapy candidates in many tumors^26, 28^. Nevertheless, molecular mechanisms underlying penile cancer remain poorly understood. Here, an integrative study was performed with four molecular levels to investigate penile carcinoma. *AR*, *BIRC5*, *DNMT3B*, *ERBB4*, *FGFR1*, *PML*, *PPARG*, *RB1* and *STAT1* genes were highlighted as potential driver candidates. In addition, 40 miRNAs, including hsa-miR-130b and hsa-miR-320, were associated with the regulation of these genes.

Recently, McDaniel *et al*.^23^ reported somatic variants in 60 PeCa from 43 patients using a panel with 126 potentially actionable genes. The authors reported non-synonymous mutations covering well-described cancer related genes, including *CDKN2A*, *TP53*, *PIK3CA*, *MYC* and *BRAF*. In addition to the somatic variants, genomic profile was also investigated. In accordance to our data, *RB1* gains and *AR*, *FGFR1* and *PPARG* losses were previously reported. Ali *et al*.^24^ described genomic variants of *AR* and *RB1* genes using a panel of 236 cancer-related genes in 20 PeCa. We also reported significant low levels of *AR* expression (P < 0.001) and four overexpressed miRNAs (hsa-miR-31-5p, hsa-miR-34a- 5p, hsa-miR-205-5p and hsa-miR-185-5p) predicted to regulate this gene^22^. In the present study, *AR* and *RB1* genes were identified as potential driver candidates, harboring genomic and epigenetic alterations that are consistent with the transcriptomic profile. Overall, these findings pointed out that multiple genetic events in *AR* and *RB1* genes are involved in penile carcinogenesis.

The tumor ability to rapidly acquire new mutations is a major limitation of targeted-gene therapies. The accumulation of alterations in passenger genes may alter the dynamics of cancer development and explain clinical events, including unconstrained tumor growth, spontaneous regression and long periods of dormancy^3^. Based on these evidences, we mapped the modules with passenger candidates and used the accumulation frequency to identify a driver-module association that would be more critical for penile carcinogenesis. Considering the final 22 driver-module association list, a high frequency of passengers was detected in modules enriched for cell cycle and immune-inflammatory response pathways. Increased levels of *BIRC5* were associated with the regulation of the majority of these modules (16, 34, 49 and 102). This gene plays an important role in cell proliferation and apoptosis inhibition^29^. Due to the overexpression of *BIRC5* during carcinogenesis, treatment targeting this gene has been increasingly recognized as a promising therapy to various cancers^30–32^. In our study, *BIRC5* gene copy number gain and downexpression of hsa-miR-135a and hsa-miR-320 were significantly associated with increased expression levels of *BIRC5* suggesting that multiple events could be involved with the aberrant activity of this gene in penile cancer.

Although poorly investigated in PeCa, aberrant levels of miRNAs were recently reported. In 10 PeCa paired with adjacent non-tumor tissues, Zhang *et al*.^33^ reported 56 miRNAs and their targets associated with the modulation of MAPK, p53, Wnt, TGF-β and PI3K-Akt signaling pathways. A miRNA-based signature including hsa-miR-1, hsa-miR-101 and hsa-miR-204 was significantly associated with lymph node metastasis and unfavorable prognosis in 24 PeCa samples^34^. Recently, by integrating miRNA and gene expression data (23 PeCa and 12 non-neoplastic penile tissues-NPT), we identified 255 mRNAs specifically regulated by 68 miRNAs^22^. In this study, 34 of 40 differentially expressed miRNA were associated with tumor development or progression. A recent study reported hsa-miR-34a as potential therapeutic target in human cancer with an essential role in tumor cell response to chemotherapeutic agents^35^. In addition to hsa-miR-34a regulation involved in the *BCL2* activity, we found increased methylation levels of *BCL2*, suggesting its importance in penile carcinogenesis. Although not selected to validation as a top driver candidate in PeCa, *BCL2* was one of the 47 driver candidates herein described.

Effective anti-cancer immunotherapy strategies are hindered by the lack of knowledge of key driver mechanisms that contribute to tumor aggressiveness and immune system evasion. The association of multiple deregulated driver-pathways may allow the design of new strategies to target driver genes that promote cancer. A significant association of *STAT1* (logFC = 1.9; Score = 119.19; Module 38) and *PPARG* (logFC = −3.5; Score = 63.84; Module 97) with immune-inflammatory pathways was detected. Furthermore, *STAT1* copy number gain and *PPARG* loss were identified as a regulatory mechanism in combination with 11 differentially expressed miRNAs. An increased level of *STAT1* has been reported as conferring cellular resistance to DNA-damaging agents and mediating tumor growth aggressiveness^36^. *PPARG* was recognized to play an important role in the immune regulation through its ability to inhibit the activity of various transcription factors, including signal transducers and transcription activators (STATs), leading to an anti-inflammatory phenotype^37, 38^. Copy number losses and miRNA regulation in genes associated with PPARG signaling pathway have the potential to contribute to an aberrant activity of the inflammatory process in PeCa. In addition, an association between driver genes and immune-inflammatory pathways may suggest a need for novel strategies to hit druggable genes and find new routes to evade the resistance acquired by tumor cells.

Despite current advances in penile carcinomas investigation, effective markers clinically useful to identify lymph node metastasis, which increase morbidity in consequence of unnecessary inguinal lymphadenectomy, are poorly described in literature^39, 40^. In 2008, Kroon *et al*.^41^ reported a 44-probe classifier able to identify patients with lymph node metastases compared with patients with no lymph nodes involvement. However, the validation set of cases was not able to confirm the results. In a previous study focusing on aberrant copy number alteration profile in PeCa, we reported a significant association between *PPARG* loss and lymph node metastasis in 46 PeCa samples^42^. Recently, we verified that higher *MMP1* expression levels revealed to be a better predictor of lymph node metastasis than the clinical-pathological features^22^. Here, *MMP1* was one of the 47 driver genes obtained in the integrative analysis, with increased expression levels possibly associated with copy number gains and down-expression of its miRNAs regulators (hsa-let-7b, hsa-let-7c, hsa-miR-342-3 and hsa-miR-134).

The combination of different molecular mechanisms involved in the regulation of gene expression pointed out two overexpressed driver candidates, *BIRC5* (Score = 109.21) and *DNMT3B* (Score = 65.24), associated with shorter overall survival (log-rank test, P = 0.026 and P = 0.002, respectively). Despite the small number of death event in our cohort (11 patients), a multivariate analysis confirmed that *DNMT3B* overexpression was significantly associated with poor overall survival (Supplementary Table S7). Increased expression levels of *BIRC5*, a member of the inhibitor of apoptosis protein (IAP), was described in a large number of malignancies^43–45^. The protein encoded by *BIRC5* was reported to be involved in cell-cycle regulation and apoptosis by inhibiting caspase-3 and −7^46^. Both activities are associated with tumor progression and resistance to therapy, highlighting *BIRC5* as a potential therapeutical target^47, 48^.

In addition to the association of *BIRC5* increased expression levels with unfavorable prognosis in PeCa, we identified copy number gains and downexpression of its miRNAs regulators (hsa-miR-320 and hsa-miR-135a) as alternative events to alter the gene expression levels and to contribute with the penile tumorigenesis.

*DNMT3B* copy number gains and down-expression of its miRNAs regulators (hsa-let-7b, hsa-let-7c and hsa-miR-145) are able to explain the increased expression levels of this gene. DNA methyltransferase 3B participates in *de novo* DNA methylation and has been reported to be involved in multiples cancer types, including gastric and lung^49, 50^. Increased levels of *DNMT3B* and hsa-miR-145 downexpression were powerful in predicting shorter survival (P < 0.05) in endometrial carcinomas^51^. An additional evidence to highlight the importance of this gene was the association between *DNMT3B* overexpression and higher incidence of lymph node metastasis in oral squamous cell carcinomas^52^.

In conclusion, novel driver candidates associated with penile carcinogenesis were described. The multidimensional analysis was able to identify high-scored genes, including *STAT1* and *PPARG*, which have potential association with dysfunctional activity of the immune system. Higher connectivity with dysregulated modules was observed for *AR* gene. The well ranked *BIRC5* and *DNMT3B* were significantly associated with unfavorable prognosis in PeCa patients.

## Methods

### Patients

Fifty three fresh-frozen usual penile squamous cell carcinomas obtained from untreated patients who underwent tumor resection at A.C.Camargo Cancer Center (São Paulo, Brazil), Barretos Cancer Hospital (Barretos, SP, Brazil) and Medical School, UNESP (Botucatu, SP, Brazil) were included in this study. Twenty-one normal glans were obtained from autopsies. Samples were submitted to cellular macrodissection and histology confirmation. PeCa samples composed of at least 80% of malignant cells were further processed. Written informed consent was obtained from all patients or relatives. This study was approved by The Human Research Ethics Committees of the Institutions (Protocols #1230/09: A.C. Camargo Cancer Center; #363–2010: Barretos Cancer Hospital, and #501.229/2013: Faculty of Medicine, Botucatu, SP, Brazil). Twenty PeCa samples were evaluated for genome-wide copy number alteration, DNA methylation, gene expression and miRNA screening. HPV status was established for all PeCa using the Linear Array HPV Test Genotyping (Roche Molecular Diagnostics). Fifteen of 53 patients were positive for high-risk HPV (16 or 18) infection. Patients were advised of the procedures and provided written informed consent. The Human Research Ethics Committees of A.C.Camargo Cancer Center (#1230/2009), Barretos Cancer Hospital (#363/2010) and Medical School-UNESP (#501.229/2013) approved this study. Clinical data is summarized in Table 3.

**Table 3:** Clinical and histopathological features of PeCa cases (N = 53). Patients were divided into two groups – dependent (N = 20) and independent (N = 33), according to the microarray analysis.

Variable	Dependent group	Independent group
	N (%)	N (%)
*Number*	20	33
*Age (years)*		
Median (interquartile range)	54.5 (46–74)	55 (45–71)
*Follow-up (months)*		
Median (interquartile range)	8.5 (6–14)	12.7 (8–29)
*Histological grade*		
I-II	12 (60%)	23 (79.3%)
III	8 (40%)	6 (20.7%)
ND	0	4
*HPV infection*		
HPV-Positive^#^	5 (25%)	12 (36.4%)
HPV-Negative	15 (75%)	21 (63.6%)
*Lymph node metastasis*		
Presence	9 (45.0%)	11 (33.3%)
Absence	11 (55.0%)	22 (66.7%)
*Perineural Invasion*		
Presence	7 (35%)	9 (27.3%)
Absence	13 (65%)	24 (72.7%)
*Vascular Invasion*		
Presence	3 (15%)	4 (12.1%)
Absence	17 (85%)	29 (87.9%)
*T Stage*		
1–2	10 (50.0%)	24 (72.7%)
3–4	10 (50.0%)	9 (27.3%)

### Data acquisition and processing

The data used for integrative analysis were obtained from previous studies of our group^14, 22, 42^. Genome-wide copy number alteration analysis was performed using Agilent Human 4 × 44 K CGH Microarrays (Agilent Technologies)^42^. Aberrant regions were identified using Fast Adaptive States Segmentation Technique 2 (FASST2) algorithm, considering significance threshold of 1 × 10^−6^, three consecutive altered probes per segment and the average log2 ratio of +0.15 for copy gains and −0.15 for losses. Alterations detected in at least 20% of the samples were selected for the integrative analysis. Datasets are available in the Gene Expression Omnibus (GEO) database (GSE50134).

Global gene expression data were obtained using the Whole Human Genome 4 × 44 K microarray platform (Agilent Technologies) as described by Kuasne *et al*.^21^. Data processing, quality control filter and normalization were obtained with Agilent Feature Extraction Software (v. 10.1.1.1) and an in-house pipeline. Genes with a mean log2 signal ratio (Cy3/Cy5) of ≥0.6 and ≤−0.6 within a 95% confidence interval (CI) were considered differentially expressed. Datasets are available in Gene Expression Omnibus (GEO) database (GSE57955).

Genome-wide methylation was performed using the Agilent 244 K Human DNA Methylation Microarray (Agilent Technologies)^14^. Workbench Standard (Ed. 5.0.14, Agilent Technologies) software and Limma 3.30.6 method^53^ algorithm were used for data normalization (Lowess) and statistical analyses, respectively. Significant genes were selected considering P < 0.05.

Non-coding RNA (miRNA) analysis were conducted using TaqMan Human MicroRNA Assay System Set v2.0 (Applied Biosystems), as previously described^22^. Pfaffl model was used for data normalization^54^, considering MammU6, RNU44 and RNU48 as reference. Statistical analysis considered a two-sample t-test (P < 0.01 and FDR < 0.05) to select differentially miRNA expression. Target transcripts of differentially expressed miRNAs were predicted by at least six algorithms using miRWalk 2.0 software (http://www.umm.uni-heidelberg.de/apps/zmf/mirwalk/).

All experiments were performed in accordance to relevant guidelines and following manufacturer’s recommendations. Details of the labeling, hybridization and normalization of the experiments were described in the Supplemental Methods S1.

### Integrative Analysis

The integrative analysis was performed in four major steps: (1) cross-platforms combination to select the most representative candidates; (2) module-based analysis, partitioning the expression matrix in significant modules of co-expressed genes; (3) driver-module assignment, to identify regulatory modules and their condition-specific regulator and (4) enrichment analysis, to select top driver-module association. The integrative strategy was illustrated in Supplementary Fig. 1.

Differentially expressed genes (GE) were compared with genome-wide copy number alteration (CNA), methylation (Me) and miRNA (Mi) data to identify genes whose expression could be explained by aberrant genomic alterations and/or epigenetic events. The most representative candidates for module-based analysis were selected using the following formula:

$${\rm{Score}}=\sum _{k=1}^{n}{{\rm{CNA}}}_{k}{{\rm{Me}}}_{k}{{\rm{Mi}}}_{k}{{\rm{Ge}}}_{k}{\rm{\alpha }}{\rm{\beta }}$$

with α as a bonus to genes identified in at least 20% of the patients and β the bonus for event agreement. For each event concordant with the gene expression profile, an added bonus was assigned (2 for two events agreement, 3 for three events and 4 if gene expression is in accordance with the other three molecular levels). For example, one overexpressed gene mapped in an amplified region, having promoter hypomethylated and regulated by a downexpressed miRNA, has bonus 4. We considered a median value between the lowest and highest scores as cutoff to select potential driver candidates for module-based analysis. Genes with score below the cutoff were defined as potential passenger genes.

In order to iteratively infer modules where genes systematically cluster together we used a Gibbs sampling procedure^27^. Modules with less than 5 genes were filtered out. The LeMoNe algorithm^55^ was used to infer a set of regulatory programs for all selected modules assigning the set of candidate genes, previously identified as the modules’ potential regulators. Using regression tree, genes were associated to each node, composed by a set of genes having similar mean and standard deviation. A score was computed to each gene-module association and the top 1% high-scoring genes were investigated.

The modules associated with the top candidates were mapped with passenger candidates to ensure the identification of modules with accumulation of secondary alterations and possibly involved in penile carcinogenesis. Modules with more than 10% of passenger candidates were selected for an enrichment analysis using Gene Set Enrichment Analysis (GSEA) algorithm considering GO (geneontology.org/), KEGG (http://www.genome.jp/kegg/) and Reactome (http://www.reactome.org/) databases. The statistical significance of module enrichment was defined with P < 0.05. The median value between the highest and lowest score was the cutoff to select the top potential driver candidates for expression levels validation using RT-qPCR.

### Cross-validation of top driver candidates and comparison with other squamous cell carcinoma (SCC) available in TCGA

RNA-seq data of 1,423 squamous cell carcinomas samples (1,325 T and 98 NT) were retrieved from TCGA (http://tcga-data.nci.nih.gov/tcga/). A total of 397 samples were excluded for having indeterminate or non-squamous cell histology and Human Papilloma Virus (HPV) positivity. The final set of samples was composed by 1,026 patients (928 SCC HPV- and 98 NT), which included head and neck (415 T and 44 NT), cervical (12 T and 3 NT) and lung squamous cell carcinomas (501 T and 51 NT). The results obtained with the TCGA data were compared with the driver candidates selected in PeCa. Samples were obtained from “level 3”, quantified at the gene levels using RSEM (RNA-Seq by Expectation Maximization), and normalized with upper-quartile.

### Gene expression analysis by RT-qPCR

A total of 53 PeCa (33 used in the array assays) and 21 NG (18 array independent) were used for RT-qPCR (following the MIQE guideline recommendations). As previously reported^56^ *, GUSB* was selected as reference. Relative quantification of the expression levels was calculated according to Pfaffl method^54^. Non-parametric Mann-Whitney test was applied to compare tumors with NG samples according to the clinicopathological features.

### Human protein-protein interaction and enrichment analysis

The protein-protein interaction was obtained from I2D^57^ that contains 71,694 predicted interactions for human identified with high-throughput data analysis. NAViGaTOR software package (ophid.utoronto.ca/navigator) was used for visualizing and analyzing protein-protein interaction networks^58^. Molecular Signatures Database (MSigDB) (software.broadinstitute.org/gsea/msigdb) and DrugBank (http://www.drugbank.ca) were used to identify association among significant modules with specific gene families (cytokines and growth factors, transcription factors, oncogenes, tumor suppressors, homeodomain proteins, cell differentiation markers and protein kinases) and drug-target genes, respectively. Databases were consulted in October 2016.

### Statistical analysis

Statistical analysis was performed using GraphPad Prism5 and SPSS version 21.0 software, adopting Two-Tailed Test and P < 0.05 value as significant. Overall survival analysis was performed using Kaplan-Meier and log rank test. High and low transcript levels in the tumor samples were defined as superior and inferior outliers compared with NG expression levels. Cross-validation of top driver candidates and comparison with other squamous cell carcinomas (SCC) available in TCGA were conducted using R 3.3.2 software^59^ and Limma 3.30.6 method (two-tailed P < 0.05 and FDR < 0.05)^53^.

## Electronic supplementary material

Supplementary Information

## References

[1] Zhang S (2012). Discovery of multi-dimensional modules by integrative analysis of cancer genomic data. Nucleic Acids Res..

[2] Vogelstein B (2013). Cancer genome landscapes. Science..

[3] McFarland CD, Korolev KS, Kryukov GV, Sunyaev SR, Mirnya LA (2013). Impact of deleterious passenger mutations on cancer progression. Proc Natl Acad Sci USA.

[4] Budzinska MA (2016). Accumulation of Deleterious Passenger Mutations Is Associated with the Progression of Hepatocellular Carcinoma. PLoS ONE..

[5] Kristensen VN (2014). Principles and methods of integrative genomic analyses in cancer. Nat Rev Cancer..

[6] Ritchie MD (2015). Methods of integrating data to uncover genotype-phenotype interactions. Nat Rev Genet..

[7] Beck AH (2015). Open access to large scale datasets is needed to translate knowledge of cancer heterogeneity into better patient outcomes. PLoS Med..

[8] Segal E (2003). Module networks: identifying regulatory modules and their condition-specific regulators from gene expression data. Nat Genet..

[9] Bonnet E, Calzone L, Michoel T (2015). Integrative multi-omics module network inference with Lemon-Tree. PLoS Comput Biol..

[10] Madhamshettiwar PB, Maetschke SR, Davis MJ, Ragan MA (2013). RMaNI: Regulatory Module Network Inference framework. BMC Bioinformatics..

[11] Barnholtz-Sloan JS, Maldonado JL, Pow-sang J, Giuliano AR (2007). Incidence trends in primary malignant penile cancer. Urol Oncol..

[12] Hakenberg OW (2015). EAU guidelines on penile cancer: 2014 update. Eur Urol..

[13] Favorito LA (2008). Epidemiologic study on penile cancer in Brazil. Int Braz J Urol..

[14] Kuasne H, Marchi FA, Rogatto SR (2013). & de Syllos Cólus, I. M. Epigenetic mechanisms in penile carcinoma. Int J Mol Sci..

[15] IARC (2007). Human papillomaviruses. IARC Monogr Eval Carcinog Risks Hum..

[16] Alemany L (2016). Role of Human Papillomavirus in Penile Carcinomas Worldwide. European Urology..

[17] da Costa WH (2015). Prognostic factors in patients with penile carcinoma and inguinal lymph node metastasis. Int J Urol..

[18] Chiang PH, Chen CH, Shen YC (2014). Intraarterial chemotherapy as the first-line therapy in penile cancer. British Journal of Cancer..

[19] Burnett AL (2016). Penile preserving and reconstructive surgery in the management of penile cancer. Nat Rev Urol..

[20] Guimarães GC, Rocha RM, Zequi SC, Cunha IW, Soares FA (2011). Penile Cancer: Epidemiology and Treatment. Curr Oncol Rep..

[21] Kuasne H (2015). Genome-wide methylation and transcriptome analysis in penile carcinoma: uncovering new molecular markers. Clin Epigenetics..

[22] Kuasne, H. *et al*. 2017. Integrative miRNA and mRNA analysis in penile carcinomas reveals markers and pathways with potential clinical impact. *Oncotarget* (2017). 10.18632/oncotarget.14783 PMC5362487 28122331

[23] McDaniel AS (2015). Genomic Profiling of Penile Squamous Cell Carcinoma Reveals New Opportunities for Targeted Therapy. Cancer Res..

[24] Ali SM (2016). Comprehensive Genomic Profiling of Advanced Penile Carcinoma Suggests a High Frequency of Clinically Relevant Genomic Alterations. Oncologist..

[25] Feber A (2016). CSN1 Somatic Mutations in Penile Squamous Cell Carcinoma. Cancer Res..

[26] Zhang W, Edwards A, Fang Z, Flemington EK, Zhang K (2016). Integrative Genomics and Transcriptomics Analysis Reveals Potential Mechanisms for Favorable Prognosis of Patients with HPV-Positive Head and Neck Carcinomas. Sci Rep..

[27] Joshi A, Van de Peer Y, Michoel T (2008). Analysis of a Gibbs sampler method for model-based clustering of gene expression data. Bioinformatics..

[28] Yang C (2016). Integrative analysis of microRNA and mRNA expression profiles in non-small-cell lung cancer. Cancer Gene Ther..

[29] Yamamoto H, Ngan CY, Monden M (2008). Cancer cells survive with survivin. Cancer Sci..

[30] Shepelev MV (2016). hTERT and BIRC5 gene promoters for cancer gene therapy: A comparative study. Oncol Lett..

[31] Wang S (2016). Nanoparticle-mediated inhibition of survivin to overcome drug resistance in cancer therapy. J Control Release..

[32] de Jong Y (2016). Targeting survivin as a potential new treatment for chondrosarcoma of bone. Oncogenesis..

[33] Zhang L (2015). MicroRNA Expression Profile in Penile Cancer Revealed by Next-Generation Small RNA Sequencing. PLoS ONE.

[34] Hartz JM (2016). Integrated Loss of miR-1/miR-101/miR-204 Discriminates Metastatic from Nonmetastatic Penile Carcinomas and Can Predict Patient Outcome. J Urol..

[35] Li XJ, Ren ZJ, Tang JH (2014). MicroRNA-34a: a potential therapeutic target in human cancer. Cell Death and Disease..

[36] Khodarev NN, Roizman B, Weichselbaum RR (2012). Molecular Pathways: Interferon/Stat1 Pathway: Role in the Tumor Resistance to Genotoxic Stress and Aggressive Growth. Clin Can Res..

[37] Martin H (2010). Role of PPAR-gamma in inflammation. Prospects for therapeutic intervention by food components. Mutat Res..

[38] Wohlfert EA, Nichols FC, Nevius E, Clark RB (2007). Peroxisome proliferator-activated receptor gamma (PPARgamma) and immunoregulation: enhancement of regulatory T cells through PPARgamma-dependent and -independent mechanisms. J Immunol..

[39] Protzel C (2009). Lymphadenectomy in the surgical management of penile cancer. Eur Urol..

[40] Sonpavde G (2013). Penile cancer: current therapy and future directions. Ann Oncol..

[41] Kroon BK (2008). Microarray gene-expression profiling to predict lymph node metastasis in penile carcinoma. BJU Int..

[42] Busso-Lopes AF (2015). Genomic profiling of human penile carcinoma predicts worse prognosis and survival. Cancer Prev Res..

[43] Ambrosini G, Adida C, Altieri DC (1997). A novel anti-apoptosis gene, Survivin, expressed in cancer and lymphoma. Nat Med..

[44] Porebska I, Sobańska E, Kosacka M, Jankowska R (2010). Apoptotic regulators: P53 and survivin expression in non-small cell lung cancer. Cancer Genomics Proteomics..

[45] Cao L (2013). OCT4 increases BIRC5 and CCND1 expression and promotes cancer progression in hepatocellular carcinoma. BMC Cancer..

[46] Shin S (2001). An anti-apoptotic protein human survivin is a direct inhibitor of caspase-3 and -7. Biochemistry..

[47] Brun SN (2015). Survivin as a therapeutic target in Sonic hedgehog-driven medulloblastoma. Oncogene..

[48] Garg H, Suri P, Gupta JC, Talwar GP, Dubey S (2016). Survivin: a unique target for tumor therapy. Cancer Cell Int..

[49] Su X (2010). Expression pattern and clinical significance of DNA methyltransferase 3B variants in gastric carcinoma. Oncol Rep..

[50] Teneng I (2015). Global identification of genes targeted by DNMT3b for epigenetic silencing in lung cancer. Oncogene..

[51] Zhang X (2013). Down-regulation of miR-145 and miR-143 might be associated with DNA methyltransferase 3B overexpression and worse prognosis in endometrioid carcinomas. Hum Pathol..

[52] Chen WC, Chen MF, Lin PY (2014). Significance of DNMT3b in Oral Cancer. PLoS ONE.

[53] Ritchie ME (2015). Limma powers differential expression analyses for RNA-sequencing and microarray studies. Nucleic Acids Res..

[54] Pfaffl MW (2001). A new mathematical model for relative quantification in real-time RT-PCR. Nucleic Acids Res..

[55] Michoel T (2007). Validating module networks learning algorithms using simulated data. BMC Bioinformatics..

[56] Muñoz JJ (2015). Down-Regulation of SLC8A1 as a Putative Apoptosis Evasion Mechanism by Modulation of Calcium Levels in Penile Carcinoma. J Urol.

[57] Brown KR, Jurisica I (2007). Unequal evolutionary conservation of human protein interactions in interologous networks. Genome Biol..

[58] Brown KR (2009). NAViGaTOR: Network Analysis, Visualization and Graphing Toronto. Bioinformatics..

[59] R Development Core Team. R: A language and environment for statistical computing. http://www.R-project.org (2016).

