# Supplementary material for: Multidimensional integrative analysis uncovers driver candidates and biomarkers in penile carcinoma
Source: Sci Rep. 2017 Jul 27;7:6707. doi: 10.1038/s41598-017-06659-1 (PMC5532302; doi:10.1038/s41598-017-06659-1)
Supplement: Supplementary file 1 — Supplementary Information [file 41598_2017_6659_MOESM1_ESM.pdf]

Multidimensional integrative analysis uncovers driver candidates and biomarkers in penile carcinoma

Fabio Albuquerque Marchi, David Correa Martins Jr, Mateus Camargo Barros Filho, Hellen Kuasne, Ariane Fidelis Busso Lopes, Helena Brentani, Jose Carlos Souza Trindade Filho, Gustavo Cardoso Guimarães, Eliney F. Faria, Cristovam Scapulatempo-Neto, Ademar Lopes, Silvia Regina Rogatto*

SUPPLEMENTAL DATA

CONTENTS:

Supplementary Methods S1 ……………………………………………………………………..… 2

Supplementary Fig1………………………………………………………………………………… 4

Supplementary Table 1……………………………………………………………………….... 5

Supplementary Table 2……………………………………………………………………….... 12

Supplementary Table 3……………………………………………………………………….... 13

Supplementary Table 4…………………………………………………………………………. 14

Supplementary Table 5…………………………………………………………………………. 15

Supplementary Table 6…………………………………………………………………………. 16

Supplementary Table 7…………………………………………………………………………. 17

References……………………………………………………………………………………….... 17

*Corresponding author:
Silvia Regina Rogatto, Professor
Email: silvia.regina.rogatto@rsyd.dk
Department of Clinical Genetics, Vejle Hospital, SLB
Kabbeltoft 25
7100 Vejle - DK

SUPPLEMENTARY METHODS S1

Genome-wide array-CGH

Genomic data were obtained in NCBI's Gene Expression Omnibus through GEO Series (GSE50134). High-quality genomic DNA (500 ng) from 20 tumor samples and a reference sample (male genomic DNA; Promega) were labeled and co-hybridized on the Agilent Human 4x44K CGH Microarrays (Agilent Technologies), according to the manufacturer's instructions and described by Busso-Lopes et al.[1] The DNA microarray scanner (Surescan High-Resolution Technology, Agilent Technologies) and Scan Control (version 8.1, Agilent Technologies) software were used to acquire the images. The threshold of $1x10^{-6}$ and segment containing at least three consecutive altered probes were used as criterion to identify copy-number alterations. An average log2 ratio of +0.15 was used for copy number gain, +0.6 for high copy-number gains and -0.15 and -1.0 to define losses and homozygous deletions, respectively. The Nexus Copy Number software (version 6.0, Biodiscovery Inc.) was used for data analysis considering the Fast Adaptive States Segmentation Technique 2 (FASST2) algorithm and the Significance Testing for Aberrant Copy-number (STAC) as statistical methods to identify copy number alterations. Altered genes detected in at least 20% of the samples were selected for the integrative analysis.

DNA methylation microarray

The Methyl-CpG immunoprecipitation microarray (MCIp-chip) protocol (Gebhard et al, 2016) was used to investigate methylated CpG-rich sequences (MethylMiner Methylated DNA Enrichment, Invitrogen Life Technologies). The enrichment of methylated sequences was performed in a subset of 20 penile cancers (PeCa) and four normal glans (NG). Genome-wide DNA methylation assay was performed using the 244K Human DNA Methylation Microarray (Agilent Technologies) platform, as described by Kuasne et al.[2] Data normalization (Lowess) (Workbench Standard, Ed. 5.0.14, Agilent Technologies) and Limma algorithm was used for statistical analyses. Differentially methylated genes between PeCa and NG samples were identified using random-variance t-test. Two tailed P value < 0.05 and False Discovery Ratio (FDR) ≤ 0.05 were considered to be statistically significant.

miRNA Expression Analysis

Macrodissected fresh frozen tissues (>80% of tumor cells) were used to obtain total RNA. A set of pre-defined primers (Megaplex RT primers ™, Pool A, Applied Biosystems) was used for cDNA synthesis, following the manufacturer's recommendation. TaqMan Human MicroRNA Assay System Set v2.0 (Applied Biosystems) was used for miRNA expression analysis as described by Kuasne et al [3]. Pfaffl [4] model was used for data normalization, considering MammU6, RNU44 and RNU48 as references. miRNAs with reduced levels detected in more than 20% of samples from each comparison were removed from the statistical analysis. Two-sample T-Test was used to compare the sample groups (two-tailed P-value<0.01 and FDR< 5% as significant) using BRB-ArrayTools (version 4.4.0). Target transcripts of differentially expressed miRNAs were predicted by at least six of 12 algorithms (miRWalk, MicroT4, miRanda, miRBridge, miRDB, miRMap, miRNAMap, PICTAR2, PITA, RNA22, RNAhybrid and Targetscan) using miRWalk 2.0 software (http://www.umm.uni-heidelberg.de/apps/zmf/mirwalk/).

Transcriptome Profile

Microarray data were obtained from the Gene Expression Omnibus database (GSE57955). Twenty PeCa (labeled with Cy3) and a pool of five normal glans obtained from necropsies (labeled with Cy5) were evaluated using the Whole Human Genome 4×44K microarray platform (Agilent Technologies)[2], following the manufacturer's recommendations. Data acquisition and normalization (Lowess) were obtained with DNA microarray scanner and Feature Extraction Software (v. 10.1.1.1), respectively. A filter to remove probes with extreme values (gIsFeatNonUnifOL, rIsFeatNonUnifOL, gIsBGNonUnifOL, rIsBGNonUnifOL, gIsFeatPopnOL, rIsFeatPopnOL, gIsBGPopnOL and rIsBGPopnOL) and not captured (gIsFound and rIsFound) was applied in both channels. Probes with emission near to background (gIsWellAboveBG and rIsWellAboveBG) or in negative controls range (gIsInNegCtrlRange and rIsInNegCtrlRange) in both channels had values changed to "0". Genes with a mean log2 signal ratio (Cy3/Cy5) of ≥ 0.6 and ≤ -0.6 within a 95% confidence interval (CI) were considered differentially expressed.

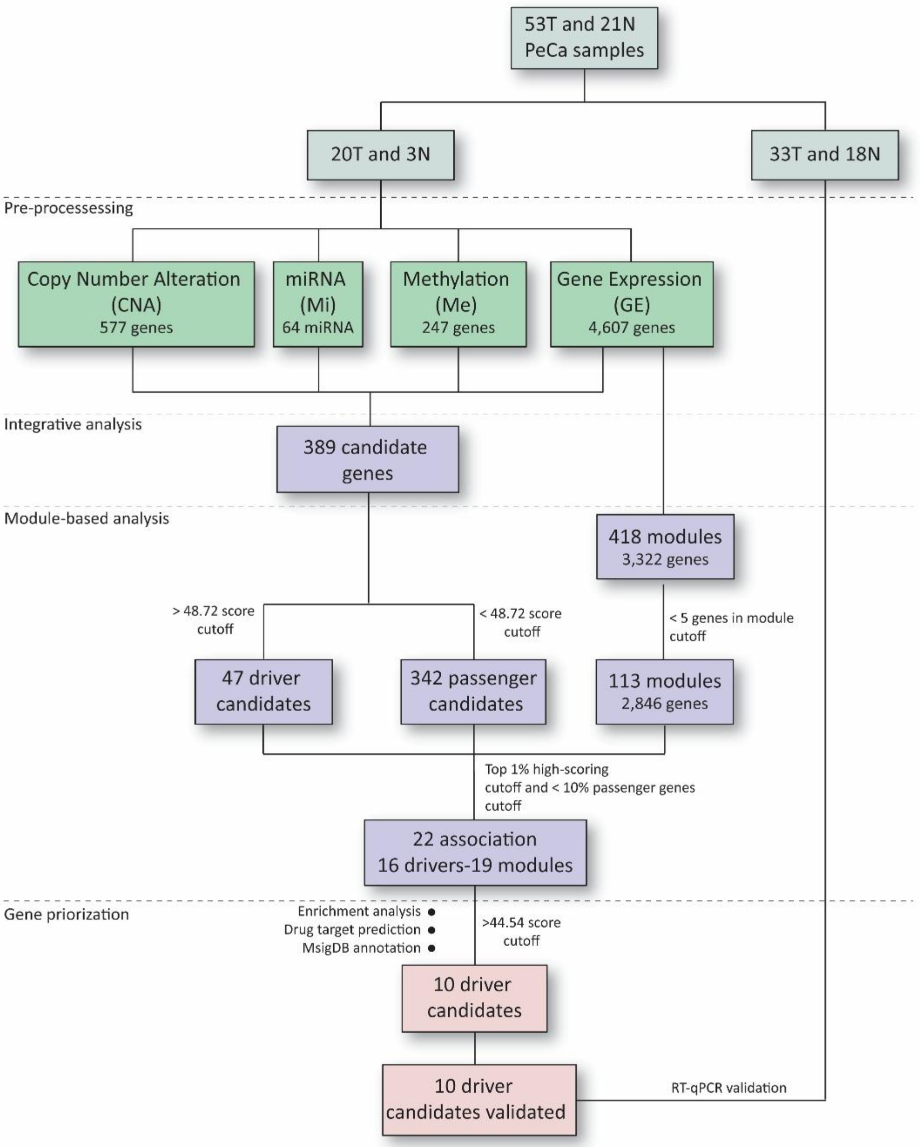

Supplementary Fig 1. Flowchart illustrating the study design. A list of 389 genes encompassing 47 potential driver candidates (defined with score above 48.72) and 342 passenger genes (defined with score below 48.72) was obtained in the first step of the integrative analysis. A total of 113 modules of co-expressed gene was identified using a Gibbs sampling and filtering out module with less than five genes. A module-based analysis performed with LeMoNe algorithm assigned the driver candidates with the remained modules. After selecting the top 1% of high-scoring driver-module association and filtering out modules with less than 10% of passenger candidates, 16 driver candidates and 19 modules were identified. Nine transcripts with score above 44.54 had the expression levels confirmed by RT-qPCR. T: Usual penile squamous cell carcinoma; N: Normal glans.

Supplementary Table S1. List of 342 passenger genes selected in the first step of the integrative analysis. Genes with score below 48.72, a median value between the lowest (4.11) and highest (101.56) score among the 389 candidates, were classified as passenger and used to map the modules of co-expressed genes. A predominance of genes mapped in chromosomes 3 and 8 was observed (54 and 51 genes, respectively).

Gene	Chromosome	CNA	FC Gene Expression	FC Methylation	FC miRNA	Score
XRN1	3	0.76	1.42		-5.88	48.38
FBXL2	3	-0.59	-1.61		5.79	47.93
CNOT7	8	-0.50	-0.90		6.35	46.49
KCNE4	2		-4.73	1.72	7.36	41.43
ZNF596	8	-0.54	-1.09		5.13	40.54
RGMB	5	-0.40	-0.96		5.28	39.84
SCARA5	8		-4.88	1.63	5.99	37.51
PDGFRL	8	-0.43	-2.94		2.87	37.45
SCARA3	8	-0.52	-1.97		3.74	37.41
ZDHHC23	3	0.42	1.17		-4.55	36.81
ICA1L	2		-2.92	1.39	7.78	36.25
CD96	3	0.43	1.18		-4.06	34.06
PODXL2	3	0.48	1.20		-3.87	33.32
PAM	5		-1.77	1.71	7.39	32.60
GRID1	10	-0.34	-2.10		2.88	31.94
PALM	19	-0.42	-2.87	2.03		31.92
TRAK2	2		-1.55	1.61	7.29	31.35
ZBTB47	3		-3.60	1.71	5.07	31.12
DCBLD2	3		1.88	-1.61	-6.86	31.06
SV2B	15		-2.73	1.51	5.89	30.41
CPD	17		0.92	-1.46	-7.67	30.13
DOCK1	10		-0.98	1.86	7.18	30.06
GRK5	10		-2.91	1.64	5.13	29.04
NOS3	7		-0.91	1.63	7.07	28.83
PARD3B	2		-2.96	1.65	4.73	28.01
STX1A	7		0.89	-1.41	-6.92	27.69
ADARB2	10		-1.85	1.93	5.40	27.54
FZD8	10		-1.52	1.44	6.13	27.24
FRZB	2		-7.26		6.31	27.12
ABLIM3	5		-1.37	1.62	5.87	26.56
MYRIP	3		-6.01		7.19	26.39
PPP1R3B	8		-1.85	1.34	5.56	26.24
PPP1R3C	10		-5.35		7.44	25.57
TAPT1	4		-1.34	1.24	5.92	25.50
CASC4	15		-1.57	1.76	5.14	25.42
WFS1	4		-1.63	2.04	4.68	25.04
FRMD4A	10		-1.76	1.74	4.68	24.54
CA2	8		3.74		-8.45	24.38
CACNB2	10		-4.49		7.58	24.14
CXCL12	10		-4.63		7.41	24.07

CDH19	18		-6.23		5.80	24.05
EREG	4		5.12		-6.71	23.65
NRP1	10		-1.41	1.38	4.95	23.22
MAL2	8		3.28		-8.22	23.00
TRIM14	9		2.57		-8.72	22.59
GLDN	15		-4.19		7.03	22.43
SLC35D1	1		1.88		-9.15	22.06
CD47	3		2.79	-1.25	-3.29	21.99
MYO5C	15		-2.96		7.91	21.74
PLAC1	X		2.77		-8.07	21.68
EPB41L4B	9		2.09		-8.68	21.54
PCDHB16	5		-2.13	1.99	3.06	21.54
ZBTB16	11	-0.43	-4.95			21.52
GPM6A	4		-5.10		5.60	21.40
MTDH	8		1.20		-9.49	21.38
RMND5A	2		0.92		-9.65	21.14
DLEC1	3	-0.35	-1.54	1.62		21.09
FAM83A	8		3.22		-7.30	21.04
PTPRF	1		2.02	-1.40	-3.58	21.01
IPPK	9		1.64		-8.74	20.75
TBC1D22B	6		1.16		-9.20	20.72
SDPR	2		-4.40		5.95	20.69
IVNS1ABP	1		1.81		-8.44	20.50
ARG2	14		1.79		-8.46	20.50
TGFBR1	9		1.41		-8.82	20.46
OSBPL2	20		0.92		-9.25	20.34
DUSP10	1		1.75		-8.41	20.32
EFHA2	8	-0.66	-4.40			20.23
EDARADD	1		1.88		-8.21	20.18
BTD	3	-0.42	-1.32	1.61		20.11
DOK6	18		-3.19		6.86	20.10
NLGN4X	X		-2.46		7.55	20.03
SENP5	3		1.59		-8.42	20.02
FAM91A1	8		1.27		-8.72	19.98
CYTL1	4		-2.96		7.03	19.97
PPP3CC	8	-0.39	-1.67	1.25		19.88
PRICKLE2	3		-2.26		7.67	19.85
TPD52	8		1.48		-8.42	19.80
KCNQ3	8		0.74	-1.57	-4.27	19.74
FADD	11		2.54		-7.34	19.74
C20orf197	20		1.57		-8.28	19.69
FAM124B	2		-2.24		7.55	19.58
ERC2	3		-2.95		6.83	19.56
VCPIP1	8		1.15		-8.60	19.52
ZAK	2		-2.22		7.49	19.41
RABL3	3		1.75		-7.95	19.40
LRP1B	2		-2.61		7.07	19.36
SST	3		1.54		-8.10	19.28
PDE1A	2		-4.15		5.46	19.22
C3orf52	3		2.94		-6.59	19.06

TOPBP1	3	1.01		-8.46	18.94
RPS14	5	-1.17	1.33	3.78	18.84
E2F1	20	2.29		-7.11	18.80
SLC44A1	9	1.11		-8.28	18.79
RALA	7	1.55		-7.84	18.78
NOL4	18	-1.87		7.50	18.73
STRN	2	1.00		-8.36	18.72
ZNF706	8	1.24		-8.12	18.72
RRS1	8	0.93		-8.41	18.68
TNFSF15	9	1.21		-8.13	18.68
RALGPS2	1	1.26		-8.07	18.67
ACTC1	15	-2.28		7.06	18.67
AZIN1	8	0.91		-8.42	18.66
PSMD5	9	0.82		-8.51	18.66
LAD1	1	1.98		-7.34	18.64
CTSW	11	0.91		-8.41	18.63
ACADSB	10	-2.36		6.93	18.56
MPPED2	11	-2.24		7.01	18.50
GPR64	X	-3.48		5.74	18.44
PLXNA1	3	1.70		-7.42	18.24
CACNA2D2	3	-2.04		7.06	18.19
KCTD12	13	-1.65		7.33	17.94
ATAD2	8	3.00		-5.92	17.83
FRMPD4	X	-1.39		7.52	17.82
TJP1	15	-1.46	1.41	3.06	17.81
SYNPR	3	-3.14		5.74	17.74
NBEA	13	-4.42		4.44	17.72
RNF180	5	-2.92		5.89	17.63
FRY	13	-4.66		4.08	17.48
RNF150	4	-3.26		5.46	17.43
PROM2	2	1.88		-6.83	17.43
CLK4	5	-1.68		7.03	17.42
E2F2	1	2.24		-6.42	17.32
C10orf32	10	-1.05		7.60	17.31
RDH10	8	1.48		-7.15	17.25
ATRNL1	10	-1.16		7.47	17.25
SLC31A2	9	2.14		-6.47	17.22
UBQLN1	9	1.56		-7.05	17.21
TFAP2C	20	2.62		-5.97	17.18
MYOZ3	5	-1.92		6.67	17.16
DERL1	8	2.18		-6.39	17.14
DEF6	6	1.54		-7.03	17.14
PGM2L1	11	-1.25		7.31	17.11
JAM3	11	-2.96		5.59	17.10
OSBPL3	7	2.30		-6.17	16.96
RAB25	1	2.26		-6.19	16.90
CIT	12	1.27		-7.18	16.89
SNRK	3	-2.02		6.43	16.89
SLIT3	5	-3.20		5.22	16.83
DPYSL2	8	-2.92		5.50	16.83

RAD1	5	1.17		-7.24	16.81
FSCN1	7	3.56		-4.83	16.79
MITF	3	-3.15		5.23	16.75
NOSTRIN	2	-4.14		4.21	16.70
PDE8B	5	-2.00		6.33	16.67
BCL11B	14	1.91		-6.40	16.62
PRKG1	10	-3.21		5.07	16.57
LRRK2	12	-2.33		5.95	16.56
XPR1	1	1.73		-6.55	16.55
KLHL4	X	-1.50		6.71	16.41
APPBP2	17	-1.39		6.77	16.33
RASL12	15	-3.89		4.27	16.32
GPR155	2	-1.91		6.18	16.18
CHRAC1	8	0.98		-7.09	16.13
PDGFD	11	-2.82		5.22	16.09
PRICKLE1	12	-1.08	1.36	2.87	15.95
MCM4	8	3.16		-4.75	15.82
GNPNAT1	14	2.32		-5.59	15.81
ITGB8	7	0.98		-6.91	15.78
TMEM2	9	0.85		-7.04	15.77
CTAGE5	14	1.92		-5.91	15.66
IER5	1	0.96		-6.86	15.64
C8orf4	8	-1.87		5.95	15.63
SLC1A3	5	2.09		-5.68	15.54
SLC39A9	14	1.28		-6.48	15.52
IL11	19	3.98		-3.77	15.49
MAPK14	6	1.01		-6.69	15.40
KIF14	1	3.43		-4.27	15.39
RAD54B	8	2.21		-5.40	15.22
COX6B2	19	2.46		-5.10	15.12
PTAFR	1	2.24		-5.23	14.93
CUBN	10	-1.08		6.34	14.83
BAI3	6	-3.48		3.93	14.82
PCDHB10	5	-1.93		5.46	14.77
EBF2	8	-1.63		5.72	14.71
RBL1	20	2.03		-5.25	14.58
CAPRIN1	11	0.90		-6.33	14.46
CLDN1	3	1.89		-5.32	14.43
HOMEZ	14	1.28		-5.91	14.37
STK32A	5	-0.90		6.24	14.30
NDP	X	-1.98		5.11	14.18
AKAP7	6	-1.50		5.58	14.15
KIF4A	X	3.00		-4.06	14.12
SNRPD1	18	1.12		-5.91	14.06
NELL1	11	-1.54		5.46	14.00
TYR	11	-4.72		2.28	13.99
TRIB1	8	1.78		-5.21	13.97
CHRM2	7	-2.01		4.95	13.92
LRRC14	8	1.16		-5.80	13.91
CREM	10	-0.90	1.32	2.41	13.91

FGFBP2	4		-3.32		3.61	13.85
TMED5	1		1.12		-5.79	13.82
XCL2	1		2.06		-4.80	13.71
IQCE	7		0.91		-5.91	13.64
MYO10	5		1.77		-4.93	13.40
YWHAZ	8		1.52		-5.18	13.39
ZNF197	3		-0.83		5.86	13.39
GBE1	3		-0.86		5.81	13.34
SLC25A26	3		-0.96		5.68	13.27
CCL7	17		2.10		-4.54	13.27
IGSF3	1		1.80		-4.79	13.19
PXDNL	8		1.99		-4.58	13.13
STIL	1		2.72		-3.82	13.10
WISP1	8		2.48		-4.06	13.09
WDR6	3	-0.31	-0.75		1.11	13.06
EFHB	3	-0.62	-2.64			13.03
ACOT7	1		1.82		-4.69	13.01
DPP3	11		2.62		-3.83	12.90
TAF2	8		1.10		-5.29	12.77
POP1	8		1.91		-4.43	12.69
ACTL8	1		2.06		-4.27	12.66
NEK2	1		3.60		-2.71	12.61
MPHOSPH9	12		1.12		-5.14	12.53
UBE2W	8		1.09		-5.17	12.51
SCN3B	11		-1.41		4.80	12.42
ZNF80	3		1.08		-5.12	12.41
PLAT	8	-0.61	-2.49			12.39
EPHA3	3		-3.91		2.28	12.37
YTHDF3	8		1.17		-5.00	12.35
PDE7A	8		2.29		-3.87	12.32
CHMP4C	8		2.37		-3.78	12.30
CTSL2	9		4.58	-1.56		12.28
SPP1	4	0.33	2.74			12.28
RBP1	3		1.60		-4.48	12.18
SPG20	13		-2.69		3.39	12.16
LRCH1	13		-1.43		4.63	12.11
CNTN3	3		-1.30		4.75	12.09
PVR	19		1.14		-4.88	12.06
SLC27A4	9		2.20		-3.80	12.00
RGS20	8		4.77	-1.21		11.97
MYH3	17		-3.69		2.28	11.94
CD80	3		0.87		-5.10	11.94
TOP1	20		1.03		-4.90	11.86
CHL1	3		-2.47		3.46	11.85
ETV4	17		2.26		-3.66	11.85
EPHB3	3		1.52		-4.40	11.83
IL1RAP	3		2.34		-3.57	11.82
DOCK3	3		-2.98		2.88	11.71
GFRA1	10		-1.78		4.05	11.66
NPAS2	2		0.72		-5.08	11.60

EAF2	3	1.15	1.74			11.55
GRM7	3		-1.41		4.35	11.52
ACAD9	3		0.93		-4.81	11.49
MARK3	14		1.16		-4.58	11.48
HIF1A	14		1.17		-4.57	11.48
SPTLC1	9		1.04		-4.69	11.47
PGLYRP4	1		2.22		-3.49	11.41
BRCA1	17		1.97		-3.66	11.26
PRR16	5		-0.95		4.68	11.25
KDELR2	7		1.29		-4.27	11.12
ANXA13	8		1.62		-3.86	10.97
PTPRC	1		1.64		-3.82	10.94
FASLG	1		2.31		-3.16	10.93
RAD54L	1		2.99		-2.45	10.88
CPM	12		-1.32		4.10	10.84
CDC42	1		1.50		-3.82	10.65
SP140	2		0.89		-4.37	10.52
C1RL	12		-1.11		4.14	10.50
HRSP12	8		1.37		-3.82	10.39
OGDH	7		1.00		-4.18	10.36
DBNL	7		1.05		-4.10	10.32
RAPGEF1	9		1.99		-3.12	10.22
PCNA	20		2.14		-2.90	10.08
ZBTB6	9		1.16		-3.86	10.05
ANGPTL5	11		-2.51		2.51	10.03
TOP2A	17		3.70		-1.31	10.02
ROBO4	11		-1.65		3.34	9.98
PLEKHF2	8		1.12		-3.85	9.94
RAMP1	2		-3.53	1.43		9.92
ACAN	15		-3.13		1.81	9.87
CDC25B	20		1.08		-3.80	9.76
STARD13	13		-2.14		2.74	9.75
BACE1	11		-1.48		3.39	9.73
FBXO16	8	-0.45	-1.97			9.70
SLAIN1	13		-1.97		2.88	9.69
ENTPD1	10		-1.15		3.68	9.65
RBMS3	3		-3.62		1.17	9.57
PURG	8		-0.78		3.98	9.52
GIMAP7	7		-2.45		2.28	9.47
ITGA3	17		1.56		-3.12	9.37
COL19A1	6		-1.17		3.50	9.36
MYC	8		1.13		-3.54	9.35
CARD11	7		1.77		-2.90	9.34
MMRN2	10		-2.19	2.47		9.32
CENPP	9		1.15		-3.49	9.28
PDCD6IP	3		-1.23		3.40	9.27
PLCL2	3		-1.98		2.65	9.26
NMT2	10		-1.08		3.52	9.20
MCM6	2		1.03		-3.53	9.13
GAL3ST1	22		-2.83	1.72		9.10

ZNF366	5		-2.75	1.74		8.98
SKIL	3		1.27		-3.16	8.85
C1orf131	1		1.08		-3.29	8.74
SLPI	20		2.61		-1.72	8.66
PIGS	17		0.84		-3.49	8.65
MOCS3	20		1.17		-3.16	8.65
FBXW2	9		1.12		-3.19	8.63
PPFIA2	12		-2.65		1.64	8.58
KLF2	19		-2.62	1.61		8.45
RPS6KA4	11		1.65		-2.45	8.21
TTLL3	3	-0.36	-1.69			8.20
ZNF660	3	-0.72	-1.32			8.13
PID1	2		-2.90		1.15	8.10
PCDHB15	5		-2.06	1.91		7.94
ZNF501	3		-1.95		1.95	7.81
SLC7A2	8		-1.15		2.74	7.78
GDA	9		2.45	-1.39		7.67
WSCD1	17		-1.88	1.90		7.56
STOX1	10		-2.42	1.34		7.50
IRAK1	X		1.27		-2.45	7.45
BRIP1	17		1.68		-1.93	7.22
ADCK5	8		1.12		-2.45	7.15
ZNF71	19		-1.83	1.72		7.11
ZNF25	10		-2.36		1.17	7.05
PCDHB12	5		-1.70	1.82		7.03
GRIA1	5		-0.85		2.61	6.91
MFSD7	4		-1.30	2.01		6.62
MCPH1	8	-0.41	-1.22			6.52
CAMK1D	10		-0.93		2.28	6.42
EIF1AY	Y		-1.65	1.50		6.29
PLS1	3		1.71		-1.31	6.05
HSP90AA1	14		1.26		-1.72	5.96
PIK3CB	3	0.40	1.08			5.92
ALLC	2		-1.47	1.46		5.85
ABCC2	10		-1.55	1.36		5.81
LZTFL1	3		-1.50	1.35		5.70
STK4	20		1.52		-1.31	5.67
GFM1	3	0.41	0.96			5.47
GCNT1	9		0.79		-1.93	5.43
TCP10L	21		-1.25	1.33		5.16
SNX13	7		1.25		-1.31	5.12
IFT88	13		-0.88		1.62	5.00
DLG1	3		1.15		-1.31	4.91
NTRK1	1		1.05	-1.39		4.88
VPRBP	3		-0.83	1.60		4.85
BAZ1A	14		1.13	-1.29		4.84
DYSF	2		-0.96	1.40		4.72
MLL	11		-0.86	1.41		4.54
RAB2A	8		0.93		-1.31	4.49
FLJ40852	7		-0.84	1.37		4.42

SLC25A2	5	-0.95	1.15	4.20
NRIP2	12	-0.91	1.15	4.11

LR: Log ratio; CNA: Copy Number Alteration; FC: Log transformed Fold-Change.

Supplementary Table S2. Differentially expressed miRNAs and their target driver candidates. After prediction analysis using miRWalk 2.0 database, 47 driver candidates showed expression levels regulated by at least one miRNA. Overexpressed miRNAs were predicted to regulate 36 down-expressed genes.

miRNA ID	logFC	Driver candidates
hsa-miR-31	130.89	*AR, ERBB4, IL17RD, MKRN2, SORCS3, TACC1, ZEB1*
hsa-miR-203	43.91	*AR, ARHGEF12, BCL2, CUL3, FOXP1, IGFBP5, IL17RD, ITPR1, MTUS1, PAX3, SORBS1, SOX7, ZEB1*
hsa-miR-135b	34.99	*AR, BCL2, CADM1, CUL3, ERBB4, GNG7, IL17RD, MTUS1, SORCS3, TGFBR2*
hsa-miR-200b	27.33	*ARHGEF12, BCL2, CNOT7, CUL3, ERBB4, FBXL2, FOXP1 ITPR1, LRIG1, SORCS3, TACC1, ZEB1*
hsa-miR-20a	15.46	*BCL2, CNOT7, CUL3, CUL3, IL17RD, LRIG1, OGG1, RGMB, SOX7, TACC1, TGFBR2*
hsa-miR-455-3p	12.83	*ARHGEF12, BCL2, CUL3, IGFBP5, PPP2CB, SVIL, ZEB1*
hsa-miR-142-3p	9.78	*AR, ARHGEF12, ERBB4, MTUS1, ZEB1*
hsa-miR-200c	8.36	*ARHGEF12, BCL2, CNOT7, CUL3, ERBB4, FBXL2, FOXP1, ITPR1, LRIG1, MTUS1, SORCS3, TACC1, ZEB1*
hsa-miR-148a	7.92	*ARHGEF12, CADM1, CTDSP1, CUL3, FOXP1, IGFBP5, IL17RD*
hsa-miR-130b	7.35	*AR, ARHGEF12, CNOT7, CUL3, ERBB4, FGFR1, FOXP1, IGFBP5, IL17RD, ITPR1, LRIG1, PPARG, SOX7, TACC1, TGFBR2, ZEB1*
hsa-miR-200a	7.33	*AR, CADM1, CUL3, ERBB4, FBXL2, FGFR1, FOXP1, GNG7, PAX3, RFX2, SVIL, TGFBR2, ZEB1*
hsa-miR-455-5p	6.66	*ARHGEF12, CUL3, FBXL2, IGFBP5, MYOM2, RANBP3*
hsa-miR-224	6.54	*ARHGEF12, BCL2, ITPR1, MKRN2, MTUS1, SORCS3, SOX7*
hsa-miR-21	5.68	*AR, ARHGEF12, BCL2, FBXL2, FOXP1, SOX7, TGFBR2, ZEB1*
hsa-miR-450b-5p	4.85	*AR, BCL2, CNOT7, CUL3, ERBB4, FOXP1, IGFBP5, IL17RD, MTUS1, OGG1, RGMB, SOX7, TGFBR2*
hsa-miR-106b	4.07	*BCL2, CNOT7, CUL3, FGFR1, FGFR2, IL17RD, LRIG1, OGG1, RGMB, SOX7, TACC1, TGFBR2*
hsa-miR-34a	3.87	*AR, ARHGEF12, BCL2, FGFR1, FGFR2, FOXP1, GNG7, GRID1, IGFBP5, ITPR1, MTUS1, PPARG, RGMB, SORBS1, SVIL, TGFBR2, ZEB1*
hsa-miR-181a	3.50	*AR, ARHGEF12, BCL2, CUL3, ERBB4, FOXP1, GRID1, RANBP3, RFX2, SORCS3, SOX7, TGFBR2*
hsa-miR-19a	3.12	*AR, ARHGEF12, CNOT7, CUL3, ERBB4, FOXP1, IL17RD, ITPR1, LRIG1, MTUS1, TACC1, TGFBR2*
hsa-miR-17	3.07	*BCL2, CNOT7, CUL3, ERBB4, FGFR1, FGFR2, IL17RD, LRIG1, OGG1, RGMB, SOX7, TACC1, TGFBR2*
hsa-miR-106a	2.99	*BCL2, CNOT7, CUL3, ERBB4, IGFBP5, IL17RD, LRIG1, OGG1, RGMB, SOX7, TACC1, TGFBR2*
hsa-miR-221	2.77	*BCL2, CNOT7, CUL3, ERBB4, FOXP1, ITPR1, SORCS3*
hsa-miR-20b	2.25	*BCL2, CNOT7, CUL3, ERBB4, FGFR1, FGFR2, IL17RD, LRIG1, OGG1, RGMB, SOX7, TACC1, TGFBR2*
hsa-miR-15b	2.22	*AR, ARHGEF12, BCL2, CADM1, CTDSP1, CUL3, FGFR1, GNG7, ITPR1, LRIG1, RANBP3, RGMB, TACC1*
hsa-miR-210	2.16	*CUL3, GNG7, WDR6*

hsa-miR-885-5p	-2.18	*PML*
hsa-let-7b	-2.48	*DNMT3B, MMP1, RB1, TNFSF10*
hsa-miR-320	-2.48	*BIRC5, PML, RB1, STAT1*
hsa-miR-370	-3.28	*PML*
hsa-miR-589	-3.80	*PML*
hsa-miR-135a	-5.20	*BIRC5*
hsa-miR-197	-5.34	*TNFSF10*
hsa-miR-296-5p	-5.48	*PML*
hsa-let-7c	-6.45	*DNMT3B, MMP1, RB1, TNFSF10*
hsa-miR-365	-6.53	*RB1*
hsa-miR-139-5p	-8.71	*TNFSF10*
hsa-miR-539-5p	-9.08	*STAT1*
hsa-miR-433	-9.78	*PML*
hsa-miR-422a	-11.22	*PML*
hsa-miR-145	-11.44	*DNMT3B*
hsa-miR-125a-3p	-12.63	*PML*
hsa-miR-338-3p	-13.90	*PML*
hsa-miR-342-3p	-14.16	*MMP1*
hsa-miR-1	-14.54	*PML*
hsa-miR-133b	-14.63	*MMP9, PML, TNFSF10*
hsa-miR-204	-16.69	*PML, RB1*
hsa-miR-489	-19.28	*PML*
hsa-miR-134	-23.93	*MMP1*
hsa-miR-504	-73.02	*PML*
hsa-miR-486-3p	-113.82	*PML*

FC: Log transformed Fold-Change.

Supplementary Table S3. Gene Ontology (GO) categories and the passenger genes significantly associated with 19 selected modules. The highest and lowest statistical value of each enriched module was showed in the P-value column. No significant results were observed for modules 49 (regulated by *FGFR1* and *BIRC5*) and 73 (regulated by *PAX3*). *BIRC5* was identified as the regulator of module 16 and was detected in four GO categories, which suggests its relevance to PeCa development.

GO categories	Module	P-value	Passengers
Signal transduction	102, 48, 6, 16, 11, 8 10, 2	6.60×10^{-3} - 2.62×10^{-10}	*RALA, STK4, ITGA3, UBQLN1, AKAP7, SNRK, TJP1, MYC, FBXL2, FZD8, WFS1, CD80, PSMD5, MAPK14, TOPBP1, FADS1, DUSP10, RABL3, RPS6KA4, TRIB1, BRIP1, CARD11, IVNS1ABP*
Regulation of cell proliferation	102, 11, 6, 48, 97	3.41×10^{-2} - 3.36×10^{-7}	*NELL1, CD80, SST, STK4, EPB41L4B, NDP, PLCL2, BRIP1, CARD11, EAF2, PTPRC, TRIB1*
Response to stimulus	102, 48, 6, 11, 55, 52, 16, 97	1.24×10^{-2} - 3.81×10^{-8}	*BRIP1, CAMK1D, CARD11, CD80, CHRM2, DUSP10, ETV4, FADD, FASLG, IFT88, ITGA3, IVNS1ABP, MAPK14, MLL, MYC, MYO10, NOS3, PGLYRP4, PLCL2, PLXNA1, PSMD5, PTPRC, SCARA3, SST, STK4, STX1A,*

			TRIB1, UBE2W, UBQLN1, XPR1, YWHAZ
Positive regulation of cell communication	48, 6, 11, 2, 97	$5.20x10^{-3}$ - $6.83x10^{-6}$	*STK4, YWHAZ, CARD11, PTPRC, CD80, PSMD5, STX1A, DBNL, MAPK14, PLCL2*
Vesicle activity	102, 48, 11, 2	4.67x10-4 - 3.86x10-6	*STX1A, ANXA13, RALA, UBQLN1, YWHAZ, CHMP4C, MYO10, CTSW, DYSF, APPBP2, BACE1, DBNL*
Cytoskeleton	102, 48, 72, 95	$3.01x10^{-4}$ - $3.47x10^{-6}$	*FSCN1, KIF14, KIF4A, NEK2, DEF6, EPB41L4B, MYO10, APPBP2, KLHL4, MCPH1, MYRIP, SORBS1*
Immune system process	10, 38, 11, 102, 6, 48, 2, 97	$2.94x10^{-2}$ - $3.31x10^{-14}$	*CD80, CTSW, GCNT1, PSMD5, EPHB3, ITGA3, MYO10, PGLYRP4, STK4, UBQLN1, PLCL2, SCARA3, CARD11, PTPRC, DUSP10, TRIB1*
Cell cycle	102, 48, 16, 72, 10	$2.17x10^{-3}$ - $1.18x10^{-5}$	*CHMP4C, DLG1, TAF2, MYC, KIF14, KIF4A, MCM4, NEK2, RALA, SENP5, MCPH1, NELL1, SLIT3, C8orf4*
Cell-cell adhesion	6, 48, 11, 40	$2.88x10^{-2}$ - $3.63x10^{-7}$	*CD80, EPB41L4B, ITGA3, MYO10, EPHB3, GCNT1, PCDHB12, CARD11, PTPRC*
Regulation of cell death	48, 55, 10, 52	$1.50x10^{-3}$ - $9.39x10^{-6}$	*SST, STK4, FADD, FASLG, CAMK1D, NOS3, UBQLN1, YWHAZ, ROBO4*
Regulation of cell differentiation	48, 6, 38, 34, 95, 97	$3.25x10^{-2}$ - $1.81x10^{-6}$	*ITGA3, STK4, EPHB3, NRP1, ZBTB16, MITF, PRKG1, SLIT3, PLCL2, CARD11, PTPRC*
Transcription factor binding	102, 85, 40, 6, 16, 95	$2.94x10^{-2}$ - $8.24x10^{-9}$	*ZNF71, VPRBP, TAF2, FZD8, TRAK2, WFS1, ZBTB16, FADS1, EBF2, CARD11, RPS6KA4, TRIB1*
Kinase activity	102, 48, 2, 6	$1.72x10^{-2}$ - $2.15x10^{-7}$	*CAMK1D, SNRK, VPRBP, EPHB3, STK4, MAPK14, CARD11*
Carbohydrate and catabolic process	2, 55, 85, 102	$7.46x10^{-3}$ - $1.14x10^{-8}$	*MAPK14, OGDH, ALLC, BACE1, PDE8B, CNOT7, NOS3, STK32A, ITGB8OGDH, WFS1*
Transport activity	40, 102, 48, 2, 55, 34, 11, 1, 95	$1.79x10^{-3}$ - $3.32x10^{-6}$	*SLC25A2, SLC7A2, SLC25A26, STX1A, AZIN1, KCNQ3, ABLIM3, ANXA13, RALA, UBQLN1, YWHAZ, CAMK1D, MLL, NOS3, CHMP4C, MYO10, GRM7, NRP1, SCN3B, DYSF, CTSW, ABCC2, GPM6A, KCNE4, SCARA5*

Supplementary Table S4. Enrichment analysis assessed with KEGG and Reactome databases. Twenty-seven pathways were significantly identified for nine modules (2, 6, 11, 34, 48, 52, 55, 85 and 95). Over-represented pathways included immune system, apoptosis and homeostasis pathways. Modules 2 and 48 were associated with the highest number of pathways (5 pathways), followed by module 52 (4 pathways).

Pathway	Module	P-value	Passenger
Regulation of mRNA stability by proteins that bind AU-rich elements	2	$7.39x10^{-5}$	*MAPK14*
Destabilization of mRNA by KSRP	2	$1.21x10^{-4}$	*MAPK14*
Immune system	2	$2.52x10^{-4}$	*MAPK14*

Adp signaling through P2RY1	2	$2.65x10^{-4}$	*MAPK14*
Signal amplification	2	$4.09x10^{-4}$	*MAPK14*
Axon guidance	6	$1.59x10^{-2}$	*MYO10, PLXNA1, PTPRC, RPS6KA4*
Fc gamma R-mediated phagocytosis	6	$3.09x10^{-2}$	*MYO10, PTPRC*
Adaptive immune system	11	$9.28x10^{-6}$	*CD80, PSMD5*
Regulation of ornithine decarboxylase	11	$1.27x10^{-5}$	*AZIN1, PSMD5*
Immune system	11	$2.02x10^{-5}$	*CD80, PSMD5*
Developmental biology	34	$9.44x10^{-5}$	*ABLIM3, NRP1, SCN3B*
Fc gamma R-mediated phagocytosis	48	$2.41x10^{-7}$	*MYO10*
Hemostasis	48	$3.57x10^{-6}$	*ITGA3, YWHAZ*
Platelet activation signaling and aggregation	48	$1.03x10^{-5}$	*YWHAZ*
Pathways in cancer	48	$9.00x10^{-5}$	*ITGA3, RALA, STK4*
Adaptive immune system	48	$9.77x10^{-5}$	*YWHAZ*
Apoptosis	52	$1.33x10^{-5}$	*FADD, FASLG, IL1RAP*
Extrinsic pathway for apoptosis	52	$2.03x10^{-5}$	*FADD, FASLG*
Pathways in cancer	52	$2.42x10^{-5}$	*E2F1, FADD, FASLG*
Apoptosis	52	$6.30x10^{-5}$	*E2F1, FADD, FASLG*
Hemostasis	55	$2.29x10^{-5}$	*NOS3*
Platelet homeostasis	55	$2.71x10^{-5}$	*NOS3*
Alpha linolenic acid (ALA) metabolism	85	$2.35x10^{-5}$	*FADS1*
Biosynthesis of unsaturated fatty acids	85	$8.21x10^{-5}$	*FADS1*
Insulin signaling pathway	95	$7.84x10^{-4}$	*PPP1R3C, SORBS1*

Supplementary Table S5. Cross-validation analysis using 1,423 squamous cell carcinomas available in the TCGA database. Transcript levels of 16 driver candidates were compared with RNA-seq data of head and neck (415T and 44N), cervical (12T and 3N) and lung squamous cell carcinomas (501T and 51N). HPV positive samples were removed. All genes were significantly identified in at least one tumor type, except *RB1*.

Driver candidate	FC PeCa GE	Cervical P-value	HNSCC P-value	Lung P-value	FC Cervical	FC HNSCC	FC Lung	S
AR	-4.84	$9.56x10^{-1}$	$1.96x10^{-4}$	$2.91x10^{-12}$	1.06	-4.47	-3.21	**
PPARG	-3.58	$6.51x10^{-2}$	$1.51x10^{-7}$	$5.32x10^{-17}$	14.80	-3.73	-3.92	**
PAX3	-3.39	$9.67x10^{-2}$	$5.16x10^{-1}$	$3.55x10^{-8}$	20.00	-1.25	191.76	*
ERBB4	-3.23	$1.79x10^{-1}$	$1.91x10^{-2}$	$8.77x10^{-17}$	3.17	-7.89	-9.58	**
IGFBP5	-3.13	$3.35x10^{-1}$	$7.96x10^{-3}$	$5.20x10^{-20}$	1.95	-1.92	2.68	**
FGFR1	-3.08	$1.61x10^{-2}$	$5.54x10^{-1}$	$1.87x10^{-1}$	6.01	-1.10	-1.10	*
BCL2	-2.70	$5.32x10^{-1}$	$2.75x10^{-5}$	$6.99x10^{-4}$	-1.28	-1.76	1.46	**
CUL3	-2.16	$1.19x10^{-1}$	$3.03x10^{-14}$	$9.08x10^{-1}$	1.31	-1.63	1.00	*
SOX7	-1.14	$4.22x10^{-1}$	$2.02x10^{-3}$	$1.73x10^{-6}$	-2.60	-1.43	-3.12	**
RANBP3	-0.92	$4.42x10^{-1}$	$5.67x10^{-3}$	$1.21x10^{-1}$	-1.14	1.07	1.04	*
RB1	0.92	$7.26x10^{-1}$	$2.38x10^{-1}$	$9.78x10^{-1}$	-1.20	1.06	-1.00	
TNFSF10	1.07	$1.02x10^{-1}$	$1.46x10^{-14}$	$1.27x10^{-2}$	-1.83	1.89	1.26	**

DNMT3B	1.39	$5.84x10^{-1}$	$1.02x10^{-60}$	$3.33x10^{-32}$	-1.40	5.12	7.91	**
PML	1.43	$1.57x10^{-1}$	$2.52x10^{-16}$	$1.73x10^{-2}$	-2.25	1.79	1.12	**
STAT1	1.94	$7.55x10^{-2}$	$5.04x10^{-17}$	$6.45x10^{-7}$	-2.87	2.54	1.41	**
BIRC5	3.74	$6.56x10^{-1}$	$6.86x10^{-37}$	$1.65x10^{-151}$	1.10	3.64	31.87	**

HNSCC: Head and Neck Squamous Cell Carcinoma; S: Significance; GE: Gene Expression; FC: Log transformed Fold-Change; *: Number of significant results.

Supplementary Table S6. RT-qPCR analysis performed in all samples and in microarray-independent set of cases confirmed the expression levels of the six selected transcripts. *TNFSF10* was confirmed (P<0.023) as significant in the independent set of 33 PeCa samples compared with 18 NG.

**Gene**	**All cases (N=53 PeCa and 21 NG)**			**Array-independent cases (N=33 PeCa and 18 NG)**		
	**Median NG**	**Median PeCa**	**P**	**Median NG**	**Median PeCa**	**P**
BIRC5	1.20	3,36	**<0.001**	0.70	3,03	**0.002**
DNMT3B	0.00	2,08	**<0.001**	0.00	2,05	**<0.001**
PML	0.00	2,01	**<0.001**	0.00	2,13	**<0.001**
RB1	0.40	2,38	**<0.001**	0.70	2,33	**0.003**
STAT1	-0.60	1,19	**<0.001**	-0.50	1,36	**0.020**
TNFSF10	-0.50	0,22	0.183	-0.50	0,76	**0.023**

Supplementary Table S7. Associations between clinicopathological variables and overall survival in penile carcinoma. Overall survival analysis was performed using Kaplan-Meier and log rank test and multivariable analysis with Cox Regression (including P<0.2 covariates in univariate analysis). *BIRC5* and *DNMT3B* overexpression (log-rank test, P=0.027 and P=0.002, respectively) was associated with shorter overall survival. The multivariate analysis confirmed the association of *DNMT3B* as an independent prognostic marker in PeCa (P=0.015, OR=5.4, CI=1.4-21.2).

**Variables**	**Univariate analysis**		**Multivariate analysis**	
	**HR ($CI_{95\%}$)**	**P***	**HR ($CI_{95\%}$)**	**P#**
**Age**				
<55 years	1.0		-	
≥55 years	0.32 (0.1-1.2)	0.077		
**Histological grade**				
I-II	1.0		-	
III	2.8 (0.9-9.3)	0.074		
**Vascular invasion**				
No	1.0		-	
Yes	4.0 (1.2-13.8)	0.018		
**Perineural invasion**				
No	1.0		-	
Yes	2.6 (0.8-8.4)	0.110		
DNMT3B				
Normal range	1.0		1.0	
Overexpression	6.6 (1.7-25.7)	0.002	5.4 (1.4-21.2)	0.015
BIRC5				
Normal range	1.0		-	
Overexpression	3.7 (1.1-12.9)	0.027		

HR: Hazard Ratio; $CI_{95\%}$: 95% Confidence Interval; *Log rank test; #Cox proportional hazard model

References

1. Busso-Lopes, A. F. e*t al*. Genomic profiling of human penile carcinoma predicts worse prognosis and survival. *Cancer Prev Res*. **8**, 149-56 (2015).
2. Kuasne, H. *et al*. Genome-wide methylation and transcriptome analysis in penile carcinoma: uncovering new molecular markers. *Clin Epigenetics*. **7**, 46 (2015).
3. Kuasne, H. *et al*. Integrative miRNA and mRNA analysis in penile carcinomas reveals markers and pathways with potential clinical impact. *Oncotarget.* **8,** 15294-306 (2017).
4. Pfaffl, M. W. A new mathematical model for relative quantification in real-time RT-PCR. *Nucleic Acids Res*. **29**, e45 (2001).
